# Effects of silybin supplementation on nutrient digestibility, hematological parameters, liver function indices, and liver-specific mi-RNA concentration in dogs

**DOI:** 10.1186/s12917-021-02929-3

**Published:** 2021-06-26

**Authors:** Maciej Gogulski, Adam Cieślak, Julia Grabska, Marie Ardois, Małgorzata Pomorska-Mól, Paweł A. Kołodziejski, Kacper Libera, Viola Strompfová, Małgorzata Szumacher-Strabel

**Affiliations:** 1 Department of Preclinical Sciences and Infectious Diseases, Poznań University of Life Sciences, Wołyńska 35, 60-637 Poznań, Poland; 2 University Centre for Veterinary Medicine, Poznań University of Life Sciences, Szydłowska 43, 60-637 Poznań, Poland; 3 Centre of Biosciences, Institute of Animal Physiology, Soltesovej 4-6, 040-01 Kosice, Slovakia; 4 Department of Animal Nutrition, Poznań University of Life Sciences, Wołyńska 33, 60-637 Poznań, Poland; 5 Department of Animal Physiology, Biochemistry and Biostructure, Poznań University of Life Sciences, Wołyńska 35, 60-637 Poznań, Poland

**Keywords:** Sylimarin, Supplement, Hepatoprotector, Nutrition, Neutraceuticals, Canine

## Abstract

**Background:**

Hepatopathies are an important group of disorders in dogs where proper nutritional care is crucial. Supplementation with a hepatoprotectant like silybin can improve liver function and should not interfere with nutrient digestibility. The purpose of this study was to investigate the effect of both pure silybin and commercial hepatoprotectant on nutrients digestibility, liver function indices and health status in healthy dogs (EXP1). Moreover, the second experiment (EXP2) investigated the effect of commercial hepatoprotectant on liver function tests and liver-associated miRNAs concentration in dogs with idiopathic liver disorder.

**Results:**

Nutrient digestibility was not affected by treatment in EXP1. Supplementation did alter the serum fatty acid profile, with no clinical relevance. The levels of liver markers such as ALT, AST and GGT significantly decreased. In EXP2, supplementation with commercial hepatoprotectant containing silybin improved liver function tests. A decrease was observed in liver serum markers such as ALT, AST and miR122 concentration.

**Conclusions:**

EXP1 confirmed that silybin (whether pure or as a commercial hepatoprotectant) does not interfere with digestion which subsequently exerts no detrimental effect on dogs’ health and metabolism. In EXP2, dietary supplementation with commercial hepatoprotectant containing silybin resulted in a decreased activity of serum liver markers, accompanied by a decrease in the concentration of liver-specific miRNA molecules. Liver function indices were consequently improved. Silybin supplementation can thus serve as an effective therapeutical tool in dogs with hepatopathies.

**Supplementary Information:**

The online version contains supplementary material available at 10.1186/s12917-021-02929-3.

## Background

Pet owners are nowadays more aware of, and responsible for, their animal’s welfare. Fortunately, this trend coexists with a rapid increase in the scope of veterinary services for dietary counseling [1]. Both pet owners and practitioners should know that an adequate diet is crucial for their animal’s good health [2]. The diet is more significant in case of serious diseases, where proper nutritional care may be a powerful tool to support the body’s fight against the illness. The veterinary literature describes special dietary recommendations for dogs suffering from heart [3–5], renal [6, 7] or gastrointestinal disorders [6, 8]. Dedicated feeds are often more digestible, by having a lowered or elevated concentration of particular ingredients or special additives which are often secondary plant metabolites [9–11]. These are commonly referred to as nutraceuticals, a term first defined by Stephen DeFelice in 1989 as an aliment or a food additive which may aid, prevent, or treat a condition by granting medical or health benefits [12]. Since liver disorders occur at a high prevalence, particular emphasis should be put on the nutrition of hepatopathic dogs. According to Watson [13], 12% of dogs in United Kingdom were postmortem diagnosed with chronic hepatitis. The etiology may vary from infectious agents such as canine adenovirus 1 (CAV-1) or leptospirosis, to non-infectious factors like neoplasia, poisoning, or inherited malformations [14]. Regardless of the underlying problem, appropriate feeding should be considered as an effective mean of handling liver diseases. One relevant question is whether nutraceutical supplementation further affects nutrients digestibility, which is worth considering, since patients with hepatic disorders are reported to show signs of gastrointestinal dysfunctions [15–17].

Silybin is a secondary plant metabolite exhibiting health-beneficial properties. Silibinin, composed of silybin A and B isomers, is one of the most active flavonolignans present in the extract of milk thistle (*Silybum marianum*) [10]. Along with other flavonolignans (isosilibinin, silidianin, and silicristin), it forms a complex known as silymarin. As far as we know, the literature does not discuss silybin as an antinutrient, and there has been no study of its effects on nutrient digestibility. Furthermore, no study has compared pure silybin with commercial hepatoprotectant containing silybin supplementation. For this reason, in the first experiment (EXP1) we hypothesised that the supplementation of neither pure silybin (SIL) nor commercial hepatoprotectant (HEP), containing silybin as a bioactive compound, affects nutrient digestibility in healthy dogs. Additionally, a control group (CON) of non-supplemented dogs also took part in the study. Henceforth we assumed that supplementation improves liver function, whilst not exerting a detrimental effect on general health or blood parameters. In the second experiment (EXP2), we hypothesized that HEP supplementation improves liver function in hepatopathic dogs. The main objectives of this study were thus: 1) to investigate the effects of diet supplemented with either SIL or HEP on nutrient digestibility, general health status, immunological parameters (serum cytokines, immunoglobulins, and acute phase protein concentrations) as well as liver function indices in clinically healthy dogs, and 2) to examine the effects of diet supplemented with HEP on liver function indices in dogs with hepatopathies.

## Results

### Clinical observations and mortality: EXP1

During EXP1, all dogs were healthy. No clinical symptoms nor mortality was observed.

### Clinical observations and mortality: EXP2

During EXP2, all dogs presented for veterinary consultation with at least one liver-related symptom such as decreased appetite (9/15 dogs), lethargy/depression (5/15 dogs), icterus (3/15 dogs), polyuria and polydipsia (6/15 dogs), vomiting (7/15 dogs), diarrhea (2/15 dogs). No mortality was noticed during the observation time.

### Chemical composition and fatty acid profile of the diet: EXP1

The diet chemical composition fed to the dogs in EXP1 is given in Table 1, along with its fatty acid profile.

**Table 1:** Chemical composition and fatty acid profile of the diet fed to the dogs (EXP1)

Item	Value
Gross energy (MJ/kg)	21.1
Dry matter (g/kg)	936
Organic matter (g/kg)	867
Crude protein (g/kg)	293
Crude fiber (g/kg)	62.6
Ether extract (g/kg)	138
Crude ash (g/kg)	69.0
FA (g/100 g FA)	
C14:0	1.73
C16:0	17.99
C16:1	2.97
C18:0	10.55
C18:1 *cis* 9	31.41
C18:2 *cis* 9 *cis* 12	19.83
C18:3 *cis* 9 *cis* 12 *cis* 15	5.63
C20:3n6	1.10
C20:5n3	0.60
C22:5n3	0.60
C22:6n3	0.62
Other	6.97

Abbreviations: UFA, unsaturated fatty acids; MUFA, monounsaturated fatty acids; PUFA, polyunsaturated fatty acids; MCFA, medium chain fatty acids; LCFA, long chain fatty acids; other FAs include C12:0, C14:1, C15:0, C16:1, C18:1 *cis*-11, C18:1 *cis*-15, C24:0, and C24:1

### Body weight and body condition score: EXP1

The treatment affected neither body weight (BW) nor the BCS of the studied dogs (*p*-value = 0.89). At the end of EXP1, the BW of the CON dogs was about 15.5 kg, for the HEP and SIL dogs BW was approximately 15.2 kg and 14.7 kg, respectively. The BCS was 5 throughout the whole experiment and did not differ between treatments (Table 2).

**Table 2:** BW and BCS of dogs fed diets supplemented with silybin and commercial hepatoprotectant (EXP1)

Item	CON (*n* = 18) mean	HEP (*n* = 18) mean	SIL (*n* = 18) mean	SEM	*P*
BW	15.5	15.2	14.7	0.13	NSD
BCS	5	5	5	0	NSD

Abbreviations: BW, body weight; BCS, body condition score; CON, control group; HEP, group fed diet supplemented with commercial hepatoprotectant containing silybin; NSD, not statistically different; SIL, group fed diet supplemented with pure silybin

### Apparent digestibility: EXP1

Nutrient and dry matter (DM) digestibility were not affected by the treatment, although a higher ether extract digestibility was detected in the CON group, which was not seen in HEP and SIL group (Table 3).

**Table 3:** Apparent nutrient digestibility of dogs fed diet supplemented with silybin and commercial hepatoprotectant (EXP1)

Item	CON (*n* = 18) mean	HEP (*n* = 18) mean	SIL (*n* = 18) mean	SEM	*P*
DM^4^	0.86	0.82	0.85	0.01	NSD
CP^5^	0.85	0.87	0.88	0.01	NSD
Ether extract	0.98^**a**^	0.96^**b**^	0.96^**b**^	0.01	0.02
Crude Ash	0.47	0.46	0.41	1.91	NSD

Abbreviations: CON, control group; CP, crude protein; DM, dry matter; HEP, group fed diet supplemented with commercial hepatoprotectant containing silybin; NSD, not statistically different; SIL, group fed diet supplemented with pure silybin

Superscripts ^a,b^ in the same row indicate significant differences between control versus treatment groups (*P* <  0.05)

### Hematology and serum biochemistry: EXP1

SIL and HEP had an effect on hematological parameters (Table 4). The WBC was lower in the treatment groups than in the CON group. Monocyte and eosinophil counts were lower in the HEP group than in the CON and SIL groups. Neutrophil count was higher in the CON group than in the HEP and SIL groups. The RBC, hemoglobin, and hematocrit variation were not statistically significant, and no significant differences were seen in the MCV, MCH, MCHC, or PLT between the CON and treatment groups during EXP1. All hematological parameters were within their reference ranges and supplementation did not adversely affect them.

**Table 4:** Hematology and serum biochemistry of dogs fed diets supplemented with silybin and commercial hepatoprotectant (EXP1)

Item	CON (*n* = 18) mean	HEP (*n* = 18) mean	SIL (*n* = 18) mean	SEM	*P*
WBC (× 10^9^/L)	10.5^**a**^	8.84^**b**^	9.02^**b**^	0.28	0.02
NEUT (× 10^9^/L)	6.99^**a**^	5.51^**b**^	5.37^**b**^	0.25	0.01
LYM (× 10^9^/L)	2.15	2.50	2.58	0.08	NSD
MONO (× 10^9^/L)	0.90^**a**^	0.65^**b**^	.74^**ab**^	0.04	0.03
EOS (× 10^9^/L)	0.45^**a**^	0.15^**b**^	.29^**ab**^	0.05	0.03
BASO (× 10^9^/L)	0.04	0.04	.03	0.00	NSD
RBC (× 10^12^/L)	6.81	6.79	6.92	0.08	NSD
HEM (g/L)	160	164	166	1.74	NSD
HTC (%)	0.46	0.47	0.47	0.01	NSD
MCV (fL/cell)	67.9	68.7	68.5	0.34	NSD
MCH (pg/cell)	23.5^**b**^	24.1^**a**^	24.0^**a**^	0.08	0.001
MCHC (g/L)	346	352	350	1.49	NSD
PLT (× 10^9^/L)	219	245	241	8.82	NSD
Albumin (g/L)	3.7	3.6	31.0	0.31	NSD
ALT (U/L)	37.7	39.0	36.1	1.36	NSD
Alpha-Amylase (U/L)	752^**a**^	467^**b**^	593^**b**^	31.8	< 0.001
ALP (U/L)	42.6^**a**^	35.4^**b**^	31.2^**b**^	1.29	< 0.001
AST (U/L)	28.6	32.6	32.2	0.89	NSD
Total protein (g/L)	58.2	59.6	59.4	0.55	NSD
Total bilirubin (μmol/l)	2.94^**b**^	3.39^**a**^	3.03^**ab**^	0.07	0.04
Cholesterol (mmol/L)	4.28	4.61	4.69	0.12	NSD
CK (U/L)	152	154	154	5.71	NSD
Fructosamine (μmol/l)	242^**b**^	265^**a**^	258^**ab**^	3.32	0.01
GLDH (U/L)	2.98	3.40	3.27	0.09	NSD
GLUC (mmol/L)	6.18^**a**^	5.09^**b**^	5.42^**b**^	0.11	< 0.001
GGT (U/L)	2.30^**b**^	4.08^**a**^	3.32^**a**^	0.20	< 0.001
Creatinine (μmol/l)	71.8	67.5	68.0	1.64	NSD
LDH (U/L)	157	141.1	146	12.3	NSD
Lipase (DGGR) (U/L)	58.8	68.0	76.5	3.41	NSD
Urea (mmol/L)	3.98	4.60	4.48	0.15	NSD
TG (mmol/L)	0.42^**b**^	0.59^**a**^	0.54^**a**^	0.02	< 0.001
Chloride (mmol/L)	114	112	113	0.34	NSD
Inorganic phosphorus (mmol/L)	1.31	1.52	1.52	0.05	NSD
Magnesium (mmol/L)	0.70^**b**^	0.79^**a**^	0.80^**a**^	0.01	< 0.001
Potassium (mmol/L)	4.17^**b**^	4.52^**a**^	4.54^**a**^	0.05	< 0.001
Sodium (mmol/L)	148	146	147	0.37	0.05
Calcium (mmol/L)	2.56^**a**^	2.44^**b**^	2.50^**ab**^	0.02	0.001
Albumin / globulin ratio	1.12	1.07	1.11	0.03	NSD
Globulin (g/L)	27.5	29.0	28.4	0.59	NSD

Abbreviations: CON, control group; HEP, group fed diet supplemented with commercial hepatoprotectant containing silybin; SIL, group fed diet supplemented with pure silybin; WBC, White blood cells; NEUT, neutrophils; LYM, lymphocytes; MONO, monocytes; EOS, eosinophils; BASO, basophils; RBC, red blood cells; HEM, hemoglobin; HTC, hematocrit; MCV, mean corpuscular volume; MCHC, mean corpuscular hemoglobin concentration; PLT, platelets; ALT, alanine aminotransferase; ALP, alkaline phosphatase; AST, aspartate aminotransferase; CK, creatinine kinase; GLDH, glutamate dehydrogenase; GLUC, glucose; GGT, gamma glutamyl transpeptidase; LDH, lactic dehydrogenase; TG, triglycerides; NSD, not statistically different

Superscripts ^a,b^ in the same row indicate significant differences between control versus treatment groups (*P* <  0.05)

We noted alpha-amylase, and ALP were significantly lower in the HEP and SIL groups than in CON (Table 4). On the other hand, total bilirubin was higher in the HEP group than in the CON group. Finally, the serum activity of GGT was significantly higher in the treatment groups than in the CON group.

Considering carbohydrates metabolism, we observed fructosamine level to be higher in the HEP group than in the CON group. Further, glucose concentration was higher in CON than in the treatment groups (Table 4). Additionally, taking into account lipid metabolism, triglyceride concentration was higher in the treatment groups than in the CON group.

Ionograms revealed that Mg^2+^ and K^+^ concentrations were higher in the treatment groups than in CON. The calcium concentration was however lower in the HEP group than in the CON group. Precise results are given in Table 4.

### Serum fatty acid profile: EXP1

Supplementation did affect the serum fatty acid profile of dogs, but with no clinical relevance. The concentration of C20:5 n3 and C22:0 was lower in the treatment groups than in the CON group (Table 5), while concentration of C15:0 and C17:0 was higher in the treatment groups than in CON group.

**Table 5:** Serum fatty acid profile of dogs fed diets supplemented with silybin and commercial hepatoprotectant (EXP1)

FA (g/100 g FA)	CON (*n* = 18) mean	HEP (*n* = 18) mean	SIL (*n* = 18) mean	SEM	*P*
C14:0	0.25	0.20	0.24	0.01	NSD
C15:0	0.08^**b**^	0.13^**a**^	0.10^**ab**^	0.01	0.02
C16:0	11.4	11.5	1.8	0.24	NSD
C17:0	0.30^**b**^	0.39^**a**^	0.34^**ab**^	0.01	0.04
C18:0	17.2	17.7	18.4	0.25	NSD
C20:0	0.18	0.22	0.19	0.01	NSD
C22:0	1.27^**a**^	0.84^**b**^	1.11^**a**^	0.06	< 0.001
C23:0	0.01	0.01	0.01	0.001	NSD
C24:0	0.03	0.03	0.03	0.001	NSD
C14:1	0.04	0.04	0.03	0.001	NSD
C15:1	0.12	0.12	0.11	0.01	NSD
C16:1	1.15	1.33	1.19	0.08	NSD
C17:1	0.16	0.14	0.14	0.01	NSD
C18:1 *t* 9	0.11	0.10	0.12	0.01	NSD
C18:1 *t* 11	0.28	0.29	0.32	0.01	NSD
C18:1 *cis* 9	11.4	11.2	11.4	0.42	NSD
C20:1 *trans*	0.06	0.06	0.07	0.01	NSD
C22:1 n9	0.33	0.24	0.30	0.02	NSD
C18:2 *c* 9 *c* 12	25.7	27.4	27.2	0.47	NSD
C18:3 *c* 9 *c* 12 *c* 15	0.64	0.79	0.83	0.04	NSD
C18:3 n6	0.04	0.04	0.05	0.001	NSD
C20:2	0.16	0.14	0.16	0.01	NSD
C20:3 n6	19.0	17.7	16.8	0.50	NSD
C20:4 n6	0.02	0.02	0.02	0.001	NSD
C20:5 n3	0.99^**a**^	0.58^**b**^	0.54^**b**^	0.06	< 0.001
C22:5 n3	2.01	2.72	2.30	0.13	NSD
C22:6 n3	0.04	0.04	0.04	0.01	NSD
Others	9.79	8.09	9.30	–	–
SUM	100	100	100	–	–
^1^SFA	31.6	31.5 (1.30)	32.6 (2.10)	0.43	NSD
^2^UFA	68.4	68.5 (1.30)	67.4 (2.10)	0.43	NSD
^3^MUFA	19.7	18.9 (.96)	19.2 (1.15)	0.45	NSD
^4^PUFA	48.5	49.4	48.0	0.71	NSD
n-6	45.0	45.3	44.4	0.63	NSD
n-3	3.68	4.12	3.71	0.13	NSD
n6/n3	12.4	11.0	12.3	0.37	NSD
n6 PUFA	44.7	45.2	44.1	0.63	NSD
n3 PUFA	3.68	4.12	3.71	0.13	NSD
PUFA/SFA	1.55	1.57	1.48	0.04	NSD
LNA/LA	41.8	34.9	34.6	1.92	NSD
^5^MCFA	13.2	13.4	12.7	0.30	NSD
^6^LCFA	86.6	86.4	87.1	0.31	NSD

Abbreviations: CON, control group; HEP, group fed diet supplemented with commercial hepatoprotectant containing silybin; SIL, group fed diet supplemented with pure silybin; SFA, saturated fatty acids; UFA, unsaturated fatty acids; MUFA, monounsaturated fatty acids; PUFA, polyunsaturated fatty acids; MCFA, medium chain fatty acids; LCFA, long chain fatty acids; NSD, not statistically different

Superscripts ^a,b^ in the same row indicate significant differences between control versus treatment groups (*P* <  0.05)

### Serum cytokines, immunoglobulins, and acute phase proteins: EXP1

The supplementation had no significant effect on inflammatory proteins, but IL4 concentration was significantly higher in the treatment groups than in the CON group (Table 6). IL10 concentration was higher in the SIL group than in the CON group.

**Table 6:** Serum Interleukins, immunoglobulins, and acute phase proteins in dogs fed diets supplemented with silybin and commercial hepatoprotectant (EXP1)

Item	Unit	CON (*n* = 18)	HEP (*n* = 18)	SIL (*n* = 18)	SEM	*P*
1 L-β	(pg/ml)	10.3	13.5	14.2	3.41	NSD
IL4	(pg/ml)	382^*b*^	494^*a*^	478^*a*^	17.5	< 0.01
IL6	(pg/ml)	9409	9042	8659	322.1	NSD
IL8	(pg/ml)	22.8	114.0	52.6	29.0	NSD
IL10	(pg/ml)	12.2^*b*^	19.8^*ab*^	25.3^*a*^	1.6	< 0.01
IgA	(mg/ml)	0.805	0.744	0.769	0.25	NSD
IgG	(mg/ml)	9.2	10.3	10.1	0.51	NSD
IgM	(mg/ml)	1.397	1.393	1.441	0.042	NSD
IgE	(μg/ml)	1.794	.885	2.565	0.18	NSD
Haptoglobin	(mg/ml)	0.04	0.02	0.06	0.01	NSD
CRP	(ng/ml)	624	505	681	40	NSD
SAA	(μg/ml)	18.8	16.1	15.8	3.70	NSD

Abbreviations: CON, control group; HEP, group fed diet supplemented with commercial hepatoprotectant containing silybin; SIL, group fed diet supplemented with pure silybin; CRP, C-reactive protein; NSD, not statistically different; SA, serum amyloid-A

Superscripts ^a,b^ in the same row indicate significant differences between control versus treatment groups (*P* < 0.05)

### Urine cortisol to creatinine ratio: EXP1

Supplementation did not affect urine pH, cortisol concentration, or creatinine concentration. Thus, the cortisol:creatinine ratio was not affected either (Table 7).

**Table 7:** The urine cortisol/creatinine ratio of dogs fed diets supplemented with silybin and commercial hepatoprotectant (EXP1)

Item	Unit	CON (*n* = 18) mean	HEP (*n* = 18) mean	SIL (*n* = 18) mean	SEM	*P*
pH		7.56	6.88	7.00	0.21	NSD
Cortisol	μg/dl	13.5	13.7	13.4	0.11	NSD
Creatinine	μmol/l	16,419	14,431	12,907	1017	NSD
Cortisol: creatinine	10^−6^	29.4	26.5	28.2	0.85	NSD

Abbreviations: CON, control group; HEP, group fed diet supplemented with commercial hepatoprotectant containing silybin; SIL group fed diet supplemented with pure silybin; NSD, not statistically different

### Hematology and serum biochemistry: EXP 2

Supplementation did not affect hematological parameters in dogs with hepatopathies, moreover, liver markers such as ALT, AST, and GGT significantly decreased (Table 8).

**Table 8:** Hematology and serum biochemistry of dogs with liver disorders fed diet supplemented with commercial hepatoprotectant containing silybin (EXP2)

Item	Reference value	H1 (*n* = 15)	H28 (*n* = 15)	SEM	*P*
WBC (× 10^9^/L)	6.00–12.0	11.40	14.00	1.890	NSD
NEUT (× 10^9^/L)	3.00–9.00	7.59	9.87	1.752	NSD
LYM (× 10^9^/L)	1.00–3.60	2.60	2.37	0.362	NSD
MONO (× 10^9^/L)	0.150–0.850	0.77	1.21	0.158	NSD
EOS (× 10^9^/L)	0.040–0.600	0.42	0.50	0.068	NSD
BASO (× 10^9^/L)	0.001–0.100	0.02	0.05	0.006	0.005
RBC (× 10^12^/L)	5.50–8.50	7.03	6.61	0.511	NSD
HEM (g/L)	15.0–19.0	175.80	127.50	11.659	NSD
HTC (%)	0.440–0.550	1.66	0.41	6.919	NSD
MCV (fL/cell)	6.0–77.0	71.14	63.95	2.255	NSD
MCH (pg/cell)	21.0–27.0	24.92	2.55	1.170	NSD
MCHC (g/L)	32.0–36.0	35.20	319.00	7.141	NSD
PLT (× 10^9^/L)	15.0–50.0	204.80	154.00	8.354	NSD
Albumin (g/L)	25.0–44.0	37.71	33.50	1.160	0.046
ALT (U/L)	1.00–8.0	325.44	195.35	103.233	0.012
ALP (U/L)	1.00–141.0	83.25	153.10	39.726	NSD
AST (U/L)	1.00–76.0	35.03	24.09	4.879	0.016
Total protein (g/L)	54.0–75.0	65.18	58.60	2.535	NSD
Total bilirubin (μmol/L)	0.010–4.60	3.22	3.99	0.212	0.037
Fructosamine (μmol/L)	225.0–365.0	28.93	188.0	18.167	0.035
GLDH (U/L)	0.010–1.6	22.82	13.32	4.824	0.053
GLUC (mmol/L)	3.05–6.10	5.78	5.16	0.325	NSD
GGT (U/L)	0.010–7.00	23.58	7.34	7.535	0.027
Creatinine (μmol/L)	35.0–132.0	97.51	77.65	7.163	NSD
Urea (mmol/L)	3.30–8.30	7.42	3.72	0.977	0.045
Albumin / globulin ratio	0.670–1.60	1.09	0.83	0.081	NSD
Globulin (g/L)	18.0–45.0	31.39	36.25	1.807	0.008
L- amylase (g/L)	10–1650	3.77	33.50	1.16	0.050

Abbreviations: H1, first day of supplementation; H28, twenty-eighth day of supplementation; WBC, White blood cells; NEUT, neutrophils; LYM, lymphocytes; MONO, monocytes; EOS, eosinophils; BASO, basophils; RBC, red blood cells; HEM, hemoglobin; HTC, hematocrit; MCV, mean corpuscular volume; MCH, mean corpuscular hemoglobin MCHC, mean corpuscular hemoglobin concentration; PLT, platelets; ALT, alanine aminotransferase; ALP, alkaline phosphatase; AST, aspartate aminotransferase; GLDH, glutamate dehydrogenase; GLUC, glucose; GGT, gamma glutamyl transpeptidase; NSD, not statistically different

*P*-values below 0.05 were considered statistically significant

### Serum microRNA expression: EXP1 and EXP2

**Fig. 1:**
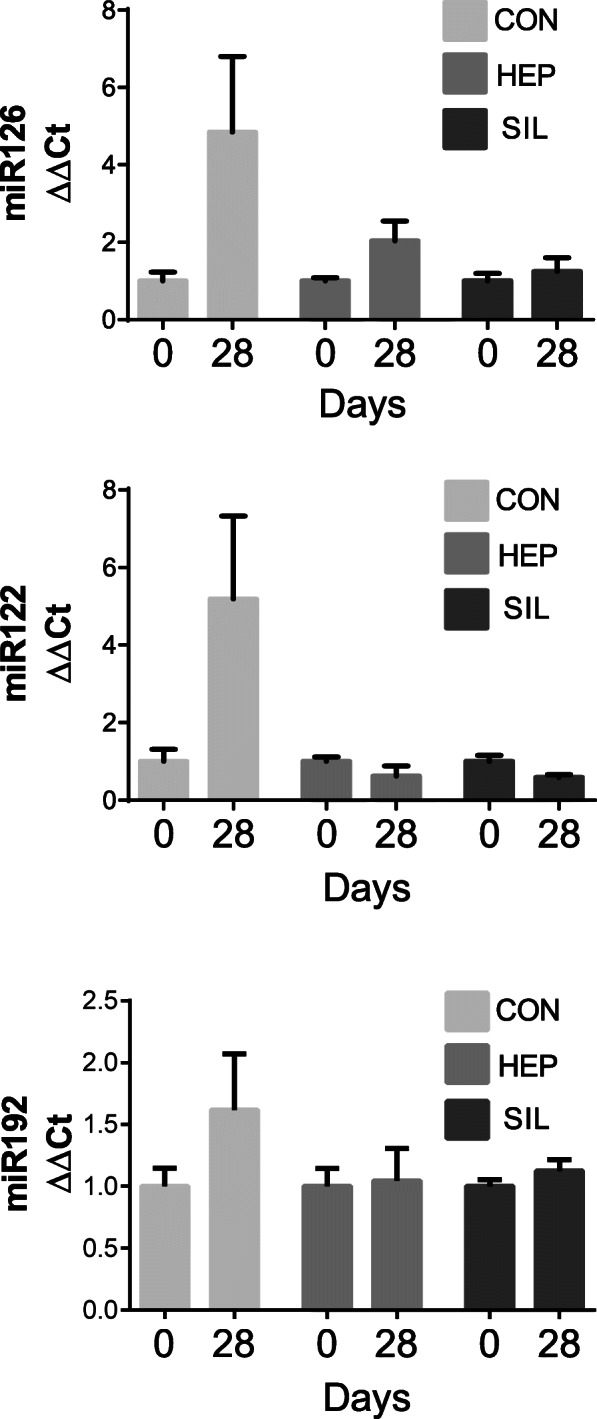
miRNA serum concentration in the healthy dogs in EXP1. CON: control group; HEP: group fed diet supplemented with commercial hepatoprotectant containing silybin; SIL: group fed diet supplemented with pure silybin. The values are expressed as mean ± SD

**Fig. 2:**
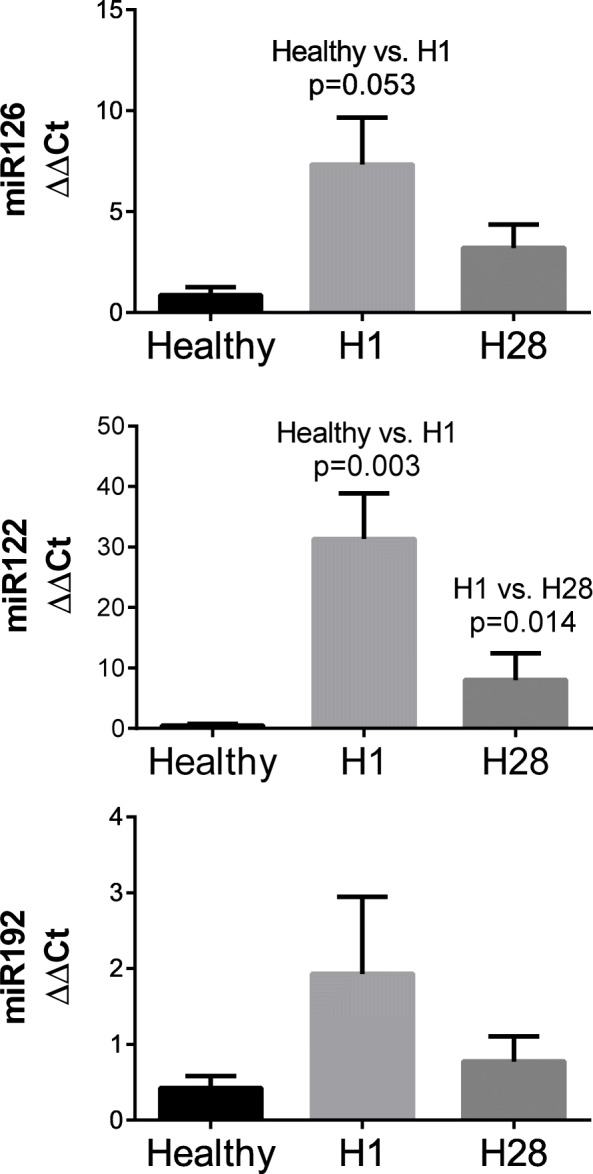
miRNA serum concentration in dogs with hepatopathies at the beginning of the supplementation with commercial hepatoprotectant containing silybin (H1) and 28 days later (H28) in EXP2. The values are expressed as mean ± SD

No difference was observed between the effects of silybin and commercial hepatoprotectant on miRNA expression in healthy dogs (Fig. 1), a significant decrease was found, after 28-day supplementation with HEP, in the expression level of miR-122. No effect of HEP on miR-192 and miR-126 was observed (Fig. 2).

## Discussion

Liver disorders are a significant group of pathologies in dogs. Patients with hepatopathies are nutritionally demanding and should be provided with high-quality feed supplemented with additives exhibiting liver-beneficial properties and not interfering with digestion. A meaningful aspect of lowered dog’s diet digestibility is the presence of antinutritional factors in it [18]. Our study (in EXP1) suggests that silybin—the active substance in silymarin—does not interfere with digestion processes. The slightly lowered ether extract digestibility in the experimental groups might be due to a negligible laxative effect (possibly caused by reduced emulsifying properties of bile containing silybin metabolites) [19] or to the occurrence of subclinical gastroenteritis, which has been reported as a rare adverse effect of silymarin [20]. However, no signs of diarrhea or other faecal consistency changes were observed in the experimental groups. Nonetheless, silybin has cholagogic properties from which we ought to expect an enhanced lipid digestion [21]. To our knowledge, no experiment has investigated the effects of silybin on nutrient digestibility in dogs. The literature does, however, discuss these effects in other animals: one report suggests that *Silybum marianum* (L.) as source of silymarin has no effect on nutrient digestibility in buffalos [22], while an experiment with broiler chickens incorporating *S. marianum* seeds into the diet resulted in increased nutrient digestibility in terms of mycotoxin-contaminated feed [23]. The present study found no clinically significant differences in hematological parameters between the dogs’ groups. Likewise, Chon and Kim [24] observed no significant differences in hematological parameters such as WBC, RBC, MCV, MCH, or MCHC between the control group and the group treated with silybin in case of dogs’ giardiasis infestation. Liver enzymes activity significantly decreased in experimental groups (both SIL and HEP), as in other studies with various liver-associated dysfunctions [25–27]. Our study shows that, in healthy dogs, liver function indices were not negatively affected by silybin supplementation. Furthermore, some reports describe a prophylactic effect of silymarin containing silybin in cats [28] and rats [29]. For example, silymarin protected the liver in healthy cats given acetaminophen. ALT/GPT, AST/GOT, ALP, and LDH did not increase, as it happened in cats given acetaminophen alone [30]. It is important to highlight that poisoning with nonsteroidal anti-inflammatory drugs (NSAIDs) occurs relatively often in small animal practice, as a result of unauthorised administration by the owner. Nevertheless, it should be kept in mind that elevated hepatic markers are not always associated with liver disease: the phenomenon can be due to a transient effect of the administration of drugs such as phenobarbital in epileptic dogs [31] or glucocorticoids [32]. The literature even describes congenital breed-related causes of elevated liver markers, like benign familial hyperphosphatasemia in Siberian huskies or increased ALP activity in Scottish terriers (where the marker may be as much as five times higher than in other breeds) [32]. The supplementation of both HEP and SIL slightly altered the serum fatty acid profile. These, statistically significant, changes were seen in the small amounts of fatty acids physiologically present in serum, and are not clinically relevant. The increase in concentration of C15:0 and C17:0 and the subsequent decrease in C20:5 n-3 may be associated with a moderate interference with lipid metabolism [33], as discussed later. To our knowledge, there are no studies to have investigated changes in the quality of the serum fatty acid profile in animals supplemented with silybin. In our study, supplementation with HEP and SIL did not affect serum cholesterol concentration, however, triglyceride concentration increased. The above results do not correlate with the study of Sun [34], who showed that in a mouse model with nonalcoholic fatty liver syndrome, silybin supplementation significantly lowered both serum and hepatic lipid accumulation. Similar results were obtained by Ramakrishnan in rats [35] with induced hepatocellular carcinoma. Moreover, the combination of silymarin and n-3 fatty acid supplementation may enhance the antihyperlipidemic effect in rats with metabolic syndrome [36]. These discrepancies with our results are probably due to the fact that different breeds and animals with induced liver disorders were used, rather than healthy animals like in our study (EXP1).

Silymarin, the source of silybin, is believed to protect against renal injury by normalizing the lipid metabolism [37]. Our study suggests that supplementation had no effect on the urine parameters, and thus on the renal function, in healthy dogs (EXP1). Silymarin is eliminated mainly by bile [38], and it does not alter urine pH, which is a significant feature in terms of urolith formation and the diagnosis of diseases (such as diabetes mellitus) by urinalysis, as it does not conceal the symptoms. Moreover, since the excretion of some drugs (such as phenobarbital or gentamycin) is related to urine pH [39], the lack of effect of both the commercial preparation and the pure silybin supplementation on urine pH seems to be advantageous.

Silymarin displays anti-inflammatory effects on T-lymphocytes in vitro [40–42]. The immunomodulatory properties of oral silymarin (silybin) in vivo in dogs have not previously been described. This study found that neither pure silybin nor commercial hepatoprotectant affected most of the immunological and inflammatory parameters. The supplementation had significant effect only on IL4 and IL10 concentration in serum. Immunoregulatory cytokines such as IL4 and IL10 have been described as exerting anti-inflammatory properties on various cell types [43–45]. The IL4 level was significantly higher in the treatment groups than in the CON group, while IL10 concentration was higher in the SIL group than in the CON group. Previous studies involving human subjects have demonstrated a significant relationship between greater hepatic inflammation and subsequent fibrosis progression [46, 47]. Thus, inflammation control may be a useful strategy against the sequelae of chronic liver disease. Here we have demonstrated that silybin has the ability to increase anti-inflammatory cytokine concentration in serum and has no effect on the proinflammatory cytokine secretion in vivo, which can be considered a positive effect. Silybin administration in dogs has been well documented as an effective therapeutical tool for different types of liver injuries, such as induced toxaemia, drug poisoning, chemotherapy, and chronic hepatitis [26–28, 48]. Several reports also describe its antiviral and antineoplastic properties in laboratory animals or cell cultures [35, 49, 50]. Similarly, in the current study, silybin supplementation improved liver function regardless of the underlying hepatic disease. We consequently observed a significant decrease in liver enzymatic markers in dogs with liver disorders (EXP2).

In recent years, in addition to traditional markers for liver diseases, such as ALT, AST, ALP and GGT, genetic markers become increasingly used, including changes in the level of microRNA [51–53]. Previous studies have shown that miR-122 and miR-126 are highly specific for the liver in dogs [52, 54, 55], while miR-192 is less specific. However, in vitro research on mice has also shown that pathological changes accompanying liver damage may be reflected in miR-192 expression [56]. We therefore decided to examine these three types of miRNA as potential markers for liver metabolism. In EXP1, we investigated whether the administration of SIL and HEP affected the liver metabolism of healthy dogs; since this study showed no negative impact, we decided to investigate the effect of HEP on animals with hepatic disorders. In EXP2, we found that HEP decreased the relative expression of miR-122 after 28 days of supplementation, and also downregulated miR-126; however, in case of miR-126, only a slight trend was observed (H1 vs. H28), which was not statistically significant (*P* = 0.241).

The usefulness of miRNAs in the diagnosis of liver diseases has been confirmed by data in the literature which show that increased miR-122 expression is noted in almost all liver diseases in dogs, such as acute and chronic hepatitis, hepatocellular carcinoma, lymphoma, and other biliary diseases like extrahepatic bile duct obstruction [51]. These results have also been confirmed by Oosthuyzen et al. [55], who showed that changes in this parameter are not associated with the breed, age, or sex of the dog, and that the number of miR-122 copies increases only during the occurrence of liver disease. They moreover demonstrated a positive correlation between miR-122 and ALT, which is one of the main markers used in the diagnosis of liver diseases [55]. Based on our results in EXP2, and due to the low specificity of changes in miR-122 in relation to various liver diseases (as shown by Dirksen et al. in 2016), we can only conclude that the organ metabolism improved [51].

The second type of miRNA investigated in our study was miR-126. Although only minor changes were observed for the HEP group (H1 vs. H28), we decided to study this type of miRNA because other studies on humans have indicated that miR-126 could also be used as a liver disease marker [56]. This was also indirectly confirmed in dogs by Dirksen et al. (2016) [51], who showed that an increased number of miR-126 copies is typical of chronic hepatitis and of other liver diseases, such as hepatocellular adenoma, hepatocellular carcinoma, or acute hepatitis. This may indicate a higher specificity of this marker in the diagnosis of liver disease in dogs. Our results showed only an increased trend in miR-126 (*P* = 0.053; healthy vs. H1), which along with the biochemical markers, may indicate that the dogs were in a transition state from the acute to the chronic phases of hepatitis. Besides it should be emphasized that, after HEP supplementation, a decrease in the number of miR-126 s (H1 vs. H28) was observed, though this change again lacked statistical significance.

Overall, our results in EXP2 showed that liver diseases were accompanied by an increase in miR-122 (H1 vs. H28), while the administration of commercial hepatoprotectant decreased it; this may indicate that treatment with HEP has a positive effect. It should be noted that there is only limited data in the literature on expression changes of miRNAs in the blood during liver diseases in dogs. We thus decided to support our research with further different diagnostic parameters. We also noted a decrease in ALT, AST, and GGT activity after administration of the commercial hepatoprotectant, which also confirms that this supplement improved liver metabolism.

The hepatoprotective properties of silymarin, containing silybin, are mainly associated with its antioxidant, antifibrotic, anti-inflammatory and cholagogic effect [14]. Moreover, silymarin accelerates liver regeneration [57]. On a molecular basis, silymarin inhibits lipid peroxidation and synthesis of reactive oxygen species. It has also been found that silymarin interacts with cell and mitochondrial membranes, modifying the flux of substances through them [14]. In a regular small animal practice, it might be challenging to accurately identify the exact liver disorder (following WSAVA guidelines) due to the lack of medical equipment and financial limitations, therefore symptomatic treatment with silybin is fully acceptable and reasonable.

An important limitation of our experiments is the fact that we did not study either the dogs used for EXP2 hormonal profile or perform a liver biopsy. Hence leading our study to be considered as a pilot, carried out following the positive result of the tested additives effect obtained during EXP1. Although we have published these results, EXP2 is not a complete study covering all aspects of canine liver disorders. We nonetheless believe that this may point to new directions of research apropos this issue. We also recognise that the EXP2 results require further research, which we plan to perform.

## Conclusion

In conclusion, we have confirmed that, in healthy dogs, supplementation with silybin, at 12.75 mg per 10 kg BW, or with a commercial hepatoprotectant containing silybin, at the same dose, does not interfere with the nutrient’s digestion, and subsequently exerts no detrimental effect on liver function indices, health, or blood parameters. In dogs with hepatopathies, supplementation with commercial hepatoprotectant containing silybin, at a dose of 12.75 mg per 10 kg BW, decreased the activity of serum liver markers, which hence was accompanied by a decrease in the concentration of liver-specific miRNA molecules (mainly miR-122). Liver function was thus improved. Overall, silybin supplementation has no adverse impact on healthy dogs and supports liver function in dogs with hepatopathies.

## Methods

### Animals and experimental design: EXP1 and EXP2

All experimental procedures were performed in accordance with the guidelines of the Local Ethical Committee for Animal Research (Ministry of Science and Higher Education, Poland) as well as in compliance with the ARRIVE guidelines. The study was approved by the statement 28/2020 of the Local Ethical Commission for Investigations on Animals in Poznań, Poznań University of Life Sciences. The dog owners gave their informed consent in writing. The research consisted of two consecutive studies. The first, EXP1, surveyed a group of eighteen healthy laboratory adult beagle dogs (*n* = 18, nine females and nine males, 2 years old). In EXP1, a 3 × 3 Latin square design with 3 treatments (CON, HEP, SIL) and three periods was used. Each treatment was given to six dogs (three females and three males) in a given period, giving eighteen replicates. A commercial basic diet (Addvena, Poznań, Poland) composed of lamb (including fresh lamb meat 50%), potatoes, peas, beet pulp, animal fat, potato protein, tomato puree, dried alfalfa, flaxseed, brewer’s yeast, salmon oil, sodium phosphate dihydrate, chicory root, glucosamine, and chondroitin sulphate constituted the control diet (CON). The first experimental diet was composed of the CON supplemented with commercial hepatoprotectant containing silybin (HEP) (Hepaxan, Vebiot, Dębica, Poland), while the second diet was CON supplemented with pure silybin (SIL). The diet for both groups contained silybin, pure or as a preparation, at a dose of 12.75 mg per 10 kg body weight. EXP1 was divided into three periods, each lasting 28 days: which consisted of a 23-day adaptation phase (days 1 to 23) and a 5-day total faecal collection phase (days 24 to 28), followed by a 12-day wash-out period. The experiment lasted 108 days (each dog had three 28-day periods with 12-day wash-out periods between them). Titanium dioxide (TiO_2_) was included in the diets as a digestion marker at 0.2% of the diet. The analysed crude nutrients concentration in the diets and dietary fatty acid profiles are presented in Table 1. Each dog was housed individually in a kennel that enabled social contact among animals, was fed twice a day and had free access to water. During the adaptation phase, the dogs had access to an outside playground for exercise and socialisation. The maintenance energy requirement (MER) was estimated according to FEDIAF [58] and the diets met the MER of the dogs. Each animal taking part in the experiment was up to date on their vaccination and deworming schedules before the beginning.

EXP2 used client-owned dogs (*n* = 15), referred to the University Centre for Veterinary Medicine at Poznań University of Life Sciences, in which a hepatic disorder was diagnosed. The diagnostic process did not reveal a specific etiological agent, therefore these cases were considered idiopathic. A profile of the dogs taking part in EXP2 can be found in Supplementary Table 1. The criteria established for hepatic disorder diagnosis were a clinical demonstration of at least one of the symptoms described as most prevalent in dogs with chronic hepatitis [48], including decreased appetite, lethargy/depression, icterus, ascites, PU/PD, vomiting, diarrhea, or subsequently an increase in at least three out of these four liver markers: alanine aminotransferase (ALT), alkaline phosphatase (ALP), aspartate aminotransferase (AST), and gamma glutamyl transpeptidase (GGT). The exclusion criteria were either infectious or parasitic diseases, systemic, neurological or traumatic diseases, general food intolerance symptoms or allergy in the past. Moreover, individuals with confirmed hepatocarcinoma or other liver-associated cancers were excluded from the study. The dog owners were advised to begin supplementing their pets’ diet with commercially available preparation containing silybin (Hepaxan, Vebiot, Dębica, Poland) at the dose recommended by the manufacturer (Supplementary Table 2).

### Health status and body condition score (BCS): EXP1 and EXP2

The average weight was 18.1 kg for males and 12.9 kg for females in EXP1, and 28.7 kg for males and 22.1 kg for females in EXP 2. In EXP1, body weight was measured on days 1 and 28 of the experimental period and feed intake was recorded daily. The study supplementation duration was 28 days. In EXP2, body weight was measured at the beginning (day 1) and at end (day 28) of supplementation with the hepatoprotectant. For all dogs in EXP1, the body condition score (BCS) was assessed throughout the experimental period in line with the recommendations of the World Small Animal Veterinary Association [59]. The dogs in EXP1 and EXP2 underwent weekly check-ups consisting of physical examination, including rectal temperature measurement, mucous membrane inspection, heart and lung auscultation, and stomach palpation (abdominal examination). The dogs were assessed as clinically healthy if the physical examination revealed no pathological findings.

### Blood sample collection: EXP1 and EXP2

Blood samples were collected via cephalic venipuncture as follows:

1. EXP1: on the last day (day 28) of each treatment period at 6.00 AM.
2. EXP2: on the first day (H1) of the supplementation and 28 days later (H28).

In both EXP1 and EXP2, blood samples were collected in two vacutainer tubes. One of these contained K_3_EDTA anticoagulant and was used for hematological examination; the second tube contained serum separator gel and was used to obtain serum for biochemical and miRNA examination, fatty acid profiles, and serum interleukin, immunoglobulin, and acute phase protein analysis. Blood from the second set of tubes was left at room temperature for blood clot formation and then centrifuged at 3500 rpm for 10 min at 4 °C to obtain serum. The serum samples were transferred to Eppendorf tubes, labeled, sealed, and frozen at − 80 °C to await analysis.

### Hematology and serum biochemistry analysis: EXP1 and EXP2

CBC was performed using a Vet ABC Animal Blood Counter automatic haematological analyser (ABX, Montpellier, France) with the following parameters: red blood cell count (RBC), neutrophil count (NEUT), lymphocyte count (LYM), monocyte count (MONO), eosinophil count (EOS), basophil count (BASO), hemoglobin (HEM), hematocrit (HTC), mean corpuscular volume (MCV), mean corpuscular hemoglobin (CHC), mean corpuscular hemoglobin concentration (MCHC), white blood cells (WBC), and platelet (PLT) counts.

Biochemical analysis of ALT, ALP, AST, GGT, alpha amylase, total protein, total bilirubin, cholesterol, creatinine kinase (CK), fructosamine, glutamate dehydrogenase (GLDH), glucose (GLUC), creatinine, lactic dehydrogenase (LDH), lipase (DGGR), urea, triglycerides (TG), chloride, inorganic phosphorus, magnesium, potassium, sodium, calcium, albumin, globulin, and albumin/globulin ratio was carried out using a Dade Behring Dimension RxL analyser (Siemens Healthcare Diagnostics, Newark, DE, USA).

The reference ranges used to evaluate health status from the haematological and biochemical parameters were based on the *Merck Veterinary Manual* [60].

### Serum interleukins, immunoglobulins, and acute phase proteins analysis: EXP1

In order to determine the concentrations of selected immunological parameters, commercially available species-specific quantitative ELISA kits were used as follows: for IgA, IgG, and IgE (Wuhan Fine Biotech, China), for IgM (Signalway Antibody, MA, USA), for IL1β, IL4, IL6, IL8, and IL10 concentrations (Wuhan Fine Biotech, China), C-reactive protein (CRP) (BlueGene, Shanghai, China), serum amyloid-A (SAA) (ABclonal, Massachusetts, USA), haptoglobin (Cusabio, TX, USA). Prior to analysis, all serum samples were diluted (depending on assay range and the expected analyte concentration). For each test, serial dilutions of standards were tested in order to obtain a calibration curve, which was then computer-adjusted. From this calibration curve, the values of the unknown protein concentration samples were calculated. All analyses were performed following the manufacturer’s instructions.

### Diet and faeces sample collection: EXP1

Diet samples were collected daily during the faecal collection phase (days 24 to 28) of each period and stored at − 20 °C for further analysis. Faeces were collected daily from day 24 to 28, including at the time of daily walks. Freshly collected faeces samples were stored at − 20 °C. The total individual daily faecal output was weighed, mixed, and stored at − 20 °C to await further analysis.

### Chemical analysis of diets and faeces and digestibility calculation: EXP1

The faecal samples were dried for 72 h at 55 °C, following AOAC International guidelines [61]. The dried faeces were milled in a laboratory mill (ZM200, Retsch, Haan, Germany) using a 1 mm sieve. The chemical composition of the feed and faeces samples was analysed following AOAC [61] method no. 934.01 for dry matter, method no. 976.05 for crude protein (using a Kjel-Foss Automatic 16,210 analyser), and method no. 973.18 for crude fat (using a Soxtec System HT analyser). The apparent total tract digestibility (ATTD) of individual nutrients relative to the ratio of TiO_2_ was calculated as a percentage based on the following equation:

$$ ATTD=100-100\left(\left(\frac{TiO_{2\frac{g}{kg} diet}}{TiO_{2\frac{g}{kg} feces}}\right)x\left(\frac{nutrient_{\frac{g}{kg} feces}}{nutrient_{\frac{g}{kg} diet}}\right)\right) $$

### Dietary and serum fatty acid (FA) profile: EXP1

FA concentration was determined using a gas chromatograph [62] with some modifications. Briefly, 3 mL of 2 M NaOH solution was added to 1 g feed or 0.5 ml serum sample, respectively, in screw-cap Teflon-stoppered tubes (glass, 15 mL) for fat hydrolysis. The hydrolysed FA samples were incubated on a block heater at 90 °C for 40 min. Analysis of fatty acids methyl ester (FAME) was performed on a gas chromatograph (GC Bruker 456-GC, Billerica, MA, USA) equipped with a capillary column (100 m fused-silica, 0.25 mm i.d., 0.25 μm film thickness; Chrompack CP7420, Agilent HP). Fatty acids were identified based on their retention times and were expressed as g/100 g FA. The observed peaks were identified by comparison of their retention times with FAME standards (37 FAME Mix, Sigma Aldrich, PA, USA) using a Galaxie Work Station 10.1 (Varian, CA).

### Urine samples and urinalysis: EXP1

Free catch urine was collected on the last day of each feeding period using a Uripet urine collection device (Rocket Medical, Watford, England). Then 3 ml of urine was stored at − 20 °C and analysed for creatinine, cortisol, and pH using VetLab Station (IDEXX Poland) within two weeks of sampling.

### Serum miRNA expression: EXP1 and EXP2

The investigation into the effects of the test compounds on the expression of miRNA in blood serum was performed in two experiments: In EXP1, the effect of commercial hepatoprotectant and silybin supplementation on miRNAs expression in healthy dogs (EXP1) was examined. In EXP2, we investigated the effect of commercial hepatoprotectant on miRNA expression in dogs with liver disorders. The healthy dogs in EXP1 were used as the control group in EXP2.

MiRNA was isolated using QIAzol Lysis Reagent and miRNeasy Serum/Plasma Kit (Qiagen, Germany). Endogenous control was added to the samples during isolation (miRNeasy Serum/Plasma Spike-In Contro; Qiagen, Germany). Following extraction and elution of RNA, the samples were immediately frozen at − 80 °C. RNA content and relative purity were determined using the UV-Vis spectrophotometric method with a NanoPhotometer NP80 (Implen, Munich, Germany). Reverse transcription was performed using a miScript II RT kit, following the manufacturer’s instructions. The master mix was prepared on cooling blocks and contained 4 μl of 5× HiSpec Buffer, 2 μl of 10× Nucleics Mix, 2 μl of Reverse Transcriptase (RT), and 2 μl of RNase-free water per reaction, giving a total volume of 20 μl. 10 μl of the master mix was added to 10 μl of the total RNA extracted from serum. The reaction was performed on a Mastercycler (Eppendorf, Germany) at 37 °C for 60 min, followed by 95 °C for 5 min to inactivate the RT. The relative expression of miRNAs (miR-192, miR-122 and miR-126) was measured by real-time PCR on QuantStudio12K Flex (Applied Biosystems, USA) using specific primers (MS00029883; Cf_miR-192_1 miScript, MS00029400; Cf_miR-122_1 miScript, MS00029428; Cf_miR-126_1 miScript; Qiagen, Germany) and miScript SYBR Green PCR kit (Qiagen, Germany). SNORD72 and RNU6–2 were used as endogenous controls (MS00033719; Hs_SNORD72_11, MS00033740; Hs_RNU6–2_11; Qiagen, Germany). Relative quantification of miRNA expression was calculated with the 2-ΔΔCt method.

### Data analysis

Both data sets for EXP1 and EXP2 were analysed with IBM SPSS Statistics 24 software (SPSS Inc., Armonk, USA). For each variable in EXP1 a repeated measure analysis (one-way ANOVA) with the Tukey test as post-hoc analysis was performed. A significant value was accepted at *p* <  0.05. In both data sets Shapiro-Wilk test was applied to assess the data normality, whereas in data from EXP1 homogeneity of variance was evaluated through Levene’s test. Variables in EXP2 were compared using the dependent Student’s t-test for normally distributed variables with a significant value accepted at *p* <  0.05.

## Supplementary Information

**Additional file 1 Table S1** Characteristic of dogs involved in EXP2. **Table S2** Hepatoprotectant dosage regimen according to the manufacturer.

## Data Availability

The datasets used and analysed in the current study are available from the corresponding author on reasonable request.

## Abbreviations

ALT: alanine aminotransferase
ALP: alkaline phosphatase
AST: aspartate aminotransferase
BASO: basophils
CON: control group
CRP: C-reactive protein
EOS: eosinophils
GLDH: glutamate dehydrogenase
GLUC: glucose
GGT: gamma glutamyl transpeptidase
H1: first day of supplementation
H28: twenty-eighth day of supplementation
HEM: hemoglobin
HTC: hematocrit
HEP: group fed diet supplemented with commercial hepatoprotectant
LYM: lymphocytes
MCV: mean corpuscular volume
MCH: mean corpuscular hemoglobin
MCHC: mean corpuscular hemoglobin concentration
MONO: monocytes
NEUT: neutrophils
PLT: platelets
RBC: red blood cells
SAA: serum amyloid-A
SIL: group fed diet supplemented with pure silybin
WBC: white blood cells
