# Supplementary material for: Effects of silybin supplementation on nutrient digestibility, hematological parameters, liver function indices, and liver-specific mi-RNA concentration in dogs
Source: BMC Vet Res. 2021 Jun 26;17:228. doi: 10.1186/s12917-021-02929-3 (PMC8235871; doi:10.1186/s12917-021-02929-3)
Supplement: Supplementary file 1 — Additional file 1 Table S1 Characteristic of dogs involved in EXP2. Table S2 Hepatoprotectant dosage regimen according to the manufacturer. [file 12917_2021_2929_MOESM1_ESM.pdf]

Effects of silybin supplementation on nutrient digestibility, hematological parameters, liver function indices, and liver-specific mi-RNA concentration in dogs

Maciej Gogulski,[1,2,3] Adam Cieślak,[4] Julia Grabska,[4] Marie Ardois,[4] Małgorzata Pomorska-Mól,[1] Paweł A Kołodziejski,[5] Kacper Libera,[1] Viola Strompfová,[3] Małgorzata Szumacher-Strabel[4*]

[1]Department of Preclinical Sciences and Infectious Diseases, Poznań University of Life Sciences, Wołyńska 35, 60-637, Poznań, Poland

[2]University Center for Veterinary Medicine, Poznań University of Life Sciences, Szydłowska 43, 60-637, Poznań, Poland

[3]Centre of Biosciences, Institute of Animal Physiology, Soltesovej 4-6, 040-01 Kosice, Slovakia

[4]Department of Animal Nutrition, Poznań University of Life Sciences, Wołyńska 33, 60-637 Poznań, Poland

[5]Department of Animal Physiology, Biochemistry and Biostructure, Poznań University of Life Sciences, Wołyńska 35, 60-637 Poznań, Poland

[*]Corresponding author; E-mail: malgorzata.szumacher@up.poznan.pl;

address: Department of Animal Nutrition, Poznań University of Life Sciences, Wołyńska 33, 60-637 Poznań, Poland

email addresses:

MG: maciej.gogulski@up.poznan.pl; AC: adam.cieslak@up.poznan.pl, JG: julsgrabska@gmail.com; MA: marieardois@icloud.com; MPM: malgorzata.pomorska@up.poznan.pl; PAK: pawel.kolodziejski@up.poznan.pl; KL: kacper.libera@up.poznan.pl; VS: strompfv@saske.sk; MSS: malgorzata.szumacher@up.poznan.pl

Supplementary Table 1 Characteristic of dogs involved in EXP2

Mix[1] (6 yrs, 12 kg, M)	Caucasian Shepherd dog (2 yrs, 60 kg, M)	Yorkshire Terrier (7 mnths, 3 kg, F)
Pitbull terrier (8 mnths, 20 kg, F)	German Shepherd dog (11 yrs, 40 kg, F)	Mix[1] (8 yrs, 25 kg, M)
Golden retriever (11 yrs, 30 kg, M)	Beagle (2 yrs, 15 kg, F)	Cane Corso (5 yrs, 50 kg, F)
Whippet (5 mnths, 5 kg, F)	Bernese Mountain Dog (10 mnths, 35 kg, M)	Beagle (5 yrs, 14 kg, F)
White Swiss Shepherd Dog (7 yrs, 30 kg, F)	Pug (11 mnths, 9 kg, M)	Husky (7 yrs, 30 kg, M)

[1]Mix – Mixed-breed dog

The additional information are given in parentheses (age, body weight, sex M-male, F-female)

Supplementary Table 2 Hepatoprotectant dosage regimen according to the manufacturer

Body weight	Less than 3 kg	3 kg to 5 kg	6 kg to 10 kg	11 kg to 15 kg	16 kg to 20 kg	Above 20 kg
Daily dosage	½ of a tablet	1 tablet	1 ½ of a tablet	2 tablets	2 ½ of a tablet	3 tablets
